# Intravascular Ultrasound Guidance Is Associated with a Favorable One-Year Target Vessel Failure Rate and No Residual Myocardial Ischemia after the Percutaneous Treatment of Very Long Coronary Artery Lesions

**DOI:** 10.3390/jcdd9120445

**Published:** 2022-12-09

**Authors:** Povilas Budrys, Arvydas Baranauskas, Giedrius Davidavicius

**Affiliations:** 1 Clinic of Cardiac and Vascular Diseases, Faculty of Medicine, Vilnius University, 03101 Vilnius, Lithuania; 2 Cardiology and Angiology Center, Vilnius University Hospital Santaros Klinikos, 08410 Vilnius, Lithuania

**Keywords:** percutaneous coronary intervention, intravascular ultrasound, IVUS, fractional flow reserve, FFR, long coronary artery lesions

## Abstract

**Background:** Studies have shown that percutaneous coronary intervention (PCI) in long coronary artery lesions (≥30 mm) is associated with more frequent target vessel failure (TVF), and a significant proportion of patients have lesions that continue to induce ischemia after PCI (FFR ≤ 0.8). We investigated the impact of intravascular ultrasound (IVUS) on the functional PCI result and one-year TVF rate after the percutaneous treatment of long coronary artery lesions. **Methods:** A total of 80 patients underwent IVUS-guided PCI in long coronary artery lesions. The PCI results were validated with IVUS and FFR. Procedural outcomes were the proportion of patients with: (1) optimal physiology result (post PCI FFR value ≥ 0.9); (2) optimal anatomy result (all IVUS PCI optimization criteria met); and (3) optimal physiology and anatomy result. The clinical outcome was TVF during a one-year follow-up (target vessel (TV)-related death, TV myocardial infarction, ischemia-driven TV revascularization). **Results:** The mean stented segment length was 62 mm. The target vessel (TV) was the left anterior descending artery in 82.5% of cases. There were no patients with residual ischemia (FFR ≤ 0.8) after PCI. Optimal coronary flow (FFR ≥ 0.9) was achieved in 37.5%; optimal anatomy, as assessed by IVUS, was achieved in 68.4%; and both optimal flow and anatomy were achieved in 25% of patients. Target vessel failure during the 12-month follow-up was 2.5%. **Conclusions:** In the percutaneous treatment of very long coronary artery lesions, the use of IVUS guidance is associated with a low TVF rate during a one-year follow-up and no residual myocardial ischemia, as assessed by FFR.

## 1. Background

Fractional flow reserve (FFR) has emerged as the ‘gold standard’ technique to estimate the functional significance of coronary artery stenosis and is often used to guide treatment [1]. While the use of FFR before percutaneous coronary intervention (PCI) is increasing, post-PCI FFR measurement is not performed routinely. Clinical trials and meta-analyses have demonstrated that the higher the post-PCI FFR, the better patient’s prognosis [2,3]. Trials comparing physiology-guided (with FFR) to angiography-guided PCI have shown that performing post-PCI FFR measurement with a pullback can optimize the PCI result and reduce residual ischemia, presumptively improving patients’ outcomes [4,5]. However, a significant percentage of patients still have an ischemic FFR (≤0.8) after PCI, and achieving an optimal FFR result is often a challenge, especially in long coronary artery lesions [6]. 

Intravascular ultrasound (IVUS)-assisted PCI is superior to angiography-guided PCI in reducing the rate of target lesion failure [7,8]. However, an optimal anatomical stenting result as assessed by IVUS does not necessarily imply an optimal functional result as assessed by FFR, and neither can an optimal FFR value guarantee good stent expansion. As most clinical studies have assessed separately the FFR or IVUS impact on clinical events, it is not clear whether physiology (FFR), anatomy (IVUS), or perhaps a combination of these modalities is better at optimizing the PCI result and predicting adverse events. 

We used both tools during PCI to assess how often an optimal functional and anatomical PCI result can be achieved and to determine the rate of target vessel failure during a one-year follow-up after the percutaneous treatment of long coronary artery lesions.

## 2. Methods

**Figure 1:**
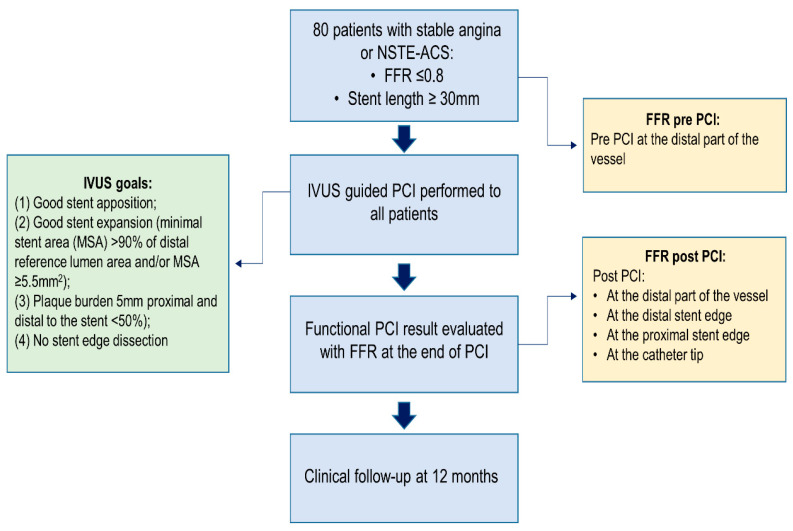
Clinical trial flowchart.

This is a single-center, prospective, observational study, performed at Vilnius University Hospital, Santaros klinikos. Eighty consecutive patients with functionally significant (FFR ≤ 0.8) lesions requiring a stent length of ≥30 mm were enrolled in the study from 1 July 2019 to 31 March 2021. All patients underwent IVUS-guided PCI and the result was assessed with both IVUS and FFR. The study flowchart is shown in Figure 1. This study complies with the Declaration of Helsinki, and the independent regional ethics committee has approved the research protocol (number of approval Nr.2019/6-1150-639). The study was conducted according to good clinical practice recommendations. All patients agreed to participate in this trial and signed an informed consent form. The study is registered on ClinicalTrials.gov (Identifier: NCT05621421).

### 2.1. Inclusion Criteria

- Chronic coronary syndrome (stable angina; staged PCI in other lesions after acute myocardial infarction with ST-segment elevation);
- Acute coronary syndrome without ST-segment elevation (unstable angina or myocardial infarction without ST-segment elevation);
- Functionally significant (FFR ≤ 0.8) lesion requiring a stent length of ≥30 mm and amenable to percutaneous coronary intervention.

### 2.2. Exclusion Criteria

- Patient’s age ≤ 18 years;
- Acute myocardial infarction with ST-segment elevation;
- Treatment with dual antiplatelet therapy contraindicated;
- Survival expectancy ≤ 1 year;
- Known allergy to sirolimus, everolimus, or zotarolimus.

### 2.3. Fractional Flow Reserve Protocol

**Figure 2:**
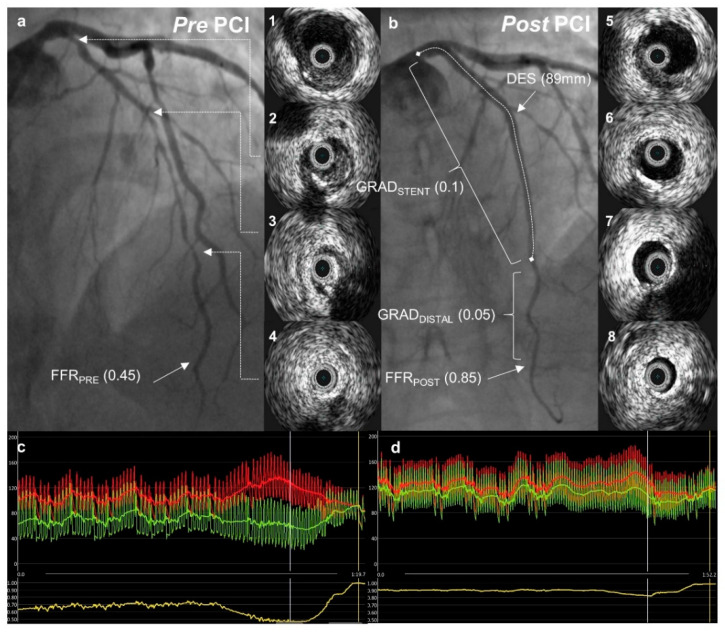
A case example. Pre- and post-percutaneous coronary intervention (PCI) angiographic images with intravascular ultrasound pictures from a corresponding left anterior descending artery segment. Fractional flow reserve curves before and after PCI are presented below. (**a**) Angiographic image demonstrating diffuse coronary artery disease in the left anterior descending artery (LAD); 1–4—intravascular ultrasound (IVUS) images from corresponding LAD segments showing predominantly soft plaque with mild calcification in mid-LAD (2, 3); (**c**) distal (Pd, green) and aortic (Pa, red) pressures with fractional flow reserve (FFR) pull-back curve (yellow) demonstrating severe ischemia in the distal LAD; (**b**) angiographic image after percutaneous coronary intervention (PCI) with an improvement in FFR to 0.85; 5–8—IVUS images from corresponding LAD segments after PCI showing acceptable stent apposition and expansion; (**d**) post-PCI FFR measurement with a pull-back demonstrating a gradual change in pressure gradient.

FFR was measured according to standard practice. Maximal hyperemia was induced with an intravenous infusion of adenosine at a rate of 140 μg/kg/min after the administration of 200 mcg of intracoronary nitroglycerine. FFR was measured using a coronary pressure wire (Abbott Vascular) and an FFR value ≤ 0.8 was considered functionally significant. The following were measured before and after PCI (Figure 2).

1. Baseline (FFR_PRE_)—defined as the evaluation of lesion significance prior to PCI, with the pressure wire sensor positioned at the distal third of the coronary artery, at least 20 mm distal to the most distal lesion. In some cases, where very distal segments were involved, the wire was positioned as far as reasonably possible.

2. Post-PCI:

- FFR_POST_—the FFR was measured in the same position as FFR_PRE_ at the end of the PCI procedure after the final IVUS run.
- FFR gradient:
  - FFR gradient across the stent (GRAD_STENT_) was defined as the difference between the FFR value just proximal to the stent and the FFR value just distal to the stent.
  - FFR gradient distal to the stent (GRAD_DISTAL_) was defined as the difference between the FFR value just distal to the stent and the FFR_POST_.

### 2.4. Intravascular Ultrasound Protocol

IVUS was performed before PCI and was used to select the stent implantation sites (optimally with a plaque burden < 50%) and stent diameter (distal external elastic membrane diameter minus 0.25 mm); Figure 2. In addition, the morphological plaque characteristics shown by IVUS guided the operators in choosing the appropriate tools for lesion preparation (semi-compliant, non-compliant, or cutting balloons). IVUS was performed using an Eagle Eye Platinum IVUS catheter (Philips, Andover MA, United States).

Operators tried to reach an optimal anatomical PCI result as assessed by IVUS if the following criteria were met: (1) good stent apposition; (2) good stent expansion (minimal stent area (MSA) > 90% of distal reference lumen area and/or MSA ≥ 5.5 mm^2^); (3) plaque burden 5 mm proximal and distal to the stent < 50%); and (4) no stent edge dissection.

After stent optimization, an IVUS run was performed. The IVUS run was considered final when further anatomical optimization was not thought to be possible.

An optimal anatomical result was defined when all four IVUS criteria were met (Figure 1).

### 2.5. PCI Procedure

The PCI was performed according to the standard practice by operators experienced in coronary physiology and intravascular imaging. Operators were encouraged to predilate all lesions. All study lesions were treated with the sirolimus (Ultimaster™), everolimus (Xience Xpedition; Promus Premier™ and Synergy™), or zotarolimus (Resolute Onyx™) drug-eluting stents. Postdilation was performed routinely. All patients received double or triple antithrombotic therapy as per European Society of Cardiology (ESC) guidelines [9,10].

### 2.6. Study Outcomes

The procedure-related outcomes were the proportions of patients with:

- Optimal physiology result (post PCI FFR value ≥ 0.9);
- Optimal anatomy result (all four IVUS PCI optimization criteria met);
- Optimal physiology and anatomy result (post PCI FFR value ≥ 0.9 and all four IVUS criteria met).

The clinical outcome was target vessel failure (TVF) during a 12-month follow-up (target-vessel-related death (TV death), target-vessel-related myocardial infarction (TV-MI), and ischemia-driven target vessel revascularization (TV-R)).

TV death—all cardiac deaths were attributed to the target vessel unless there was clear evidence indicating other reasons.

TV-MI—the presence of clinical symptoms, electrocardiographic changes, and/or imaging findings suggestive of myocardial infarction, combined with an increase in the troponin I or troponin T to a level greater than the 99th percentile of the upper normal limit.

Ischemia-driven TV-R—revascularization procedure at the previously stented vessel with >50% diameter stenosis and at least one of the following: (1) recurrence of angina; (2) positive noninvasive test; or (3) positive invasive physiologic test.

We hypothesized that post-PCI IVUS assesses mainly the target lesion, while post-PCI FFR assesses the result in the whole target vessel, including non-stented segments. We compared the clinical, procedural, and anatomical IVUS characteristics and the target vessel failure rate over a 12-month follow-up in the group that was judged to have optimal functional result (post PCI FFR ≥ 0.9) vs. patients with suboptimal functional result (post-PCI FFR < 0.9). 

### 2.7. Statistical Analysis

Continuous variables were expressed as mean (± standard deviation). Continuous variables with a normal distribution were compared using Student’s t-test, otherwise, a nonparametric Mann–Whitney U-test was used. Categorical variables were expressed as the frequency and compared using the χ^2^ test. 

## 3. Results

**Figure 3:**
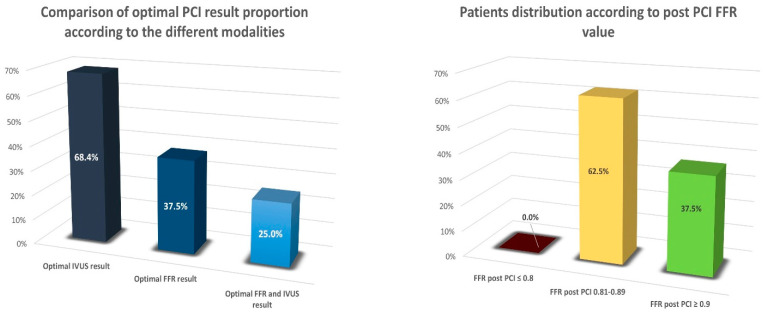
Comparison of optimal PCI result proportion according to the different modalities used and patients’ distribution according to post-PCI FFR value.

An optimal functional PCI result, as defined by a post-PCI FFR ≥ 0.9, was achieved in 37.5% of procedures. An optimal anatomical result, as defined by meeting all four IVUS criteria, was achieved in 68.4% of procedures. A total of 25% of patients had both an optimal functional and IVUS anatomical result (Figure 3). 

Baseline clinical characteristics are presented in Table 1. The mean age of all patients was 66.2 ± 8.9 years and 71.3% were males. There was no statistically significant difference between the functionally optimal vs. suboptimal result patient groups in terms of baseline clinical characteristics.

### 3.1. PCI Procedure related characteristics

PCI procedure-related characteristics are presented in Table 2. All patients underwent successful PCI. The target vessel was the left anterior descending artery (LAD) in 82.5% of patients. Every patient in the functionally suboptimal PCI group had a PCI in the LAD, while the LAD was the lesion treated in half of the patients in the FFR ≥ 0.9 group; *p* = 0.0001. 

The mean stented segment was 62.3 ± 18.0 mm, which was similar in both groups. The average stent diameter did not differ significantly between the two groups (3.4 ± 0.4 mm vs. 3.2 ± 0.3 mm, *p* = 0.14). Postdilation was performed on all stented lesions and balloon diameter was similar in both groups.

### 3.2. Fractional Flow Reserve Findings

Baseline FFR was similar among the two groups (0.64 ± 0.12 and 0.64 ± 0.09, *p* = 0.75). FFR post PCI increased to 0.94 ± 0.04 in the optimal physiology result group and to 0.86 ± 0.02 in the suboptimal physiology result group. Patients with post-PCI FFR < 0.9 had higher both distal (0.05 ± 0.03 vs. 0.02 ± 0.02, *p* = 0.0001) and trans-stent (0.08 ± 0.02 vs. 0.04 ± 0.02, *p* = 0.0001) gradients (Table 3).

### 3.3. Intravascular Ultrasound Findings

IVUS findings are presented in Table 4. Almost half of the patients had more than one post-PCI IVUS run; thus, after initial stent optimization, 40% of lesions required additional interventions in an attempt to optimize anatomical PCI results. 

Patients with suboptimal FFR results had smaller caliber distal vessels, distal reference lumen area (5.5 ± 1.7 mm^2^ vs. 6.5 ± 2.1 mm^2^, *p* = 0.03), and distal reference external elastic membrane area (8.3 ± 3.1 mm^2^ vs. 9.9 ± 3.6 mm^2^, *p* = 0.06) compared to FFR ≥ 0.9 patients. However, the plaque burden at the distal site was similar in both groups. 

Minimal stent area tended to be larger in the FFR ≥ 0.9 group (6.3 ± 1.8 mm^2^ vs. 5.6 ± 1.8 mm^2^); however, this finding did not reach statistical significance (*p* = 0.12). An optimal PCI result according to IVUS was achieved in 68.4% of patients, and this proportion was almost identical between the two groups. Patients who did not meet all four IVUS optimization goals usually failed to do so because of a ≥50% plaque burden near the stent edges.

### 3.4. Discharge Medications and Adverse Events

All patients received antithrombotic therapy (either double or triple antithrombotic therapy). Moreover, most patients were prescribed a statin, beta-blocker, ACE inhibitor, or ARB at discharge (Table 5).

There were no TV-related deaths or myocardial infarctions during the 12-month follow-up (Table 6). The target vessel failure rate was 2.5% due to stent restenosis. Both these patients were in the group that had a post PCI FFR > 0.9; one had an optimal IVUS and one a sub-optimal result, and both received PCI in non-LAD vessels.

## 4. Discussion

To our knowledge, this is the first prospective trial to use both fractional flow reserve and intravascular ultrasound in the percutaneous treatment of long coronary artery lesions.

The main findings of our study are as follows:

1. Optimal physiology PCI result (FFR ≥ 0.9) was achieved in fewer than half (37.5%) of patients; however, none of the patients had residual ischemia (FFR ≤ 0.8) after PCI.
2. An optimal anatomical PCI result (according to IVUS criteria) was achieved more often—in 68.4% of patients.
3. Only one-quarter of patients had both an optimal FFR and IVUS result.
4. Target vessel failure during the 12-month follow-up was only 2.5%. It should be underlined that this low rate of negative events was achieved in treating very long coronary artery lesions with an average stented segment length of 62 mm.

Previous trials have demonstrated that the higher the post-PCI FFR, the better the patient’s prognosis. Yet, there is no consensus regarding an optimal post-PCI FFR cutoff, which varies from >0.86 to >0.96 in other studies [4,11,12,13,14,15,16]. We have decided to use the FFR value of 0.9 as the threshold to divide patients into functionally optimal vs. suboptimal PCI result as it is the value used in the majority of trials [2,4,11,13,14,17,18].

In our sample, 37.5% of patients with very long coronary artery lesions had a post-PCI FFR value ≥ 0.9. This finding is similar to other studies. The TARGET-FFR trial used a physiology-guided PCI optimization strategy, which resulted in 38.1% of patients having post-PCI FFR ≥ 0.9; however, a significant proportion of patients (18.1%) had a post-PCI FFR ≤ 0.8, despite using FFR to optimize the procedural result [4]. On the contrary, there were no patients with residual ischemia in our study. In TARGET-FFR, the LAD was the target vessel in 57.3% of cases, compared to 82.5% in our study, and the average length of the stented segment was 31 mm, which is two times shorter compared to our trial. As LAD lesions and longer stenoses are generally associated with worse outcomes, it could be postulated that if TARGET-FFR had a similar lesion vessel distribution and stenosis length as our study, their functional PCI result could have been worse than that shown in their study. It is likely that the adjuvant use of intravascular imaging (13% in the TARGET-FFR optimization group) could have been helpful in improving the functional PCI result. Another study, which used a functional optimization strategy without the routine use of intravascular imaging, found a reduction in patients with post PCI FFR ≤ 0.8 from 21% to 8%, and 43% of patients had a post-interventional FFR > 0.9 [15]. Kimura et al. reported a retrospective analysis that included 167 patients who underwent successful PCI with IVUS stent optimization. The proportion of patients with post-PCI FFR > 0.9 was similar to ours; however, 18.6% of patients had a post-PCI FFR ≤ 0.8 [19]. That study did not have well-defined IVUS optimization criteria. Strict PCI optimization according to IVUS criteria was applied in our study, which could explain this difference in the proportion of patients with residual ischemia, especially as we were treating longer coronary artery lesions. They also found that LAD artery lesions were associated with a lower post-PCI FFR value, as in our study. 

An interesting study was performed by Hwang et al [20], where 835 patients with available post-PCI FFR measurements were evaluated. The authors concluded that different cut-off values of post-PCI FFR should be applied depending on the target vessel. They established that the optimal post-PCI FFR value cutoffs for predicting target vessel failure were 0.82 for LAD and 0.88 for non-LAD. If these cutoffs were applied to our study, an optimal functional PCI result would have been achieved in 96.3% of lesions. The adjustment to this lower cutoff value could partially explain the very low target vessel failure rate in our sample.

As per our study’s protocol, IVUS was used in all cases before and after PCI. The criteria of optimal PCI result according to IVUS were similar to those used in the ULTIMATE trial [7], except the desirable minimal stent area was larger in our study (5.5 vs. 5.0 mm^2^). We obtained an optimal IVUS result in 68.4% of patients, which is higher compared to the ULTIMATE trial, where this goal was achieved in 53% of patients. It should be noted that the ULTIMATE trial included all types of lesions, while our study’s focus was long coronary lesions. There are two randomized controlled trials wherein IVUS-guided PCI was compared to angiography-guided intervention in long coronary artery lesions. The IVUS-XPL trial randomized 1400 patients with long lesions (defined as implanted stent length ≥ 28 mm; the average length of the actually stented segment was 39 mm) to receive either IVUS-guided or angiography-guided PCI [21]. IVUS optimization criteria were not as strict as our study (MSA greater than the lumen cross-sectional area at the distal reference segment), and an optimal IVUS result was achieved in 54% of patients. Kim et al. performed a similar study, where 543 patients were randomly allocated to receive IVUS-guided or angiography-guided PCI for long coronary artery lesions (stents ≥ 28 mm in length) [22]. However, this study did not have predefined PCI optimization criteria according to IVUS, and the authors state that IVUS information could have been underutilized. The average implanted stent length was 32 mm, which is similar to the IVUS-XPL trial and considerably shorter than our study at 62 mm; therefore, our study has patients who have exceptionally long coronary artery stenoses compared to most other studies.

The cornerstone of a PCI procedure is to improve patients’ symptoms and prognosis and to avoid or minimalize the occurrence of adverse events in the future. Knowing whether optimal physiology and anatomical results are achieved at the end of PCI could give an idea of how well the treated lesion will behave in the long term. However, solely implementing physiology and imaging and trying to accomplish IVUS optimization criteria, for some patients, could be sufficient to reduce adverse events in the future. Although only 25% of patients in our sample met both optimal physiology (FFR ≥ 0.9) and optimal anatomy (all four IVUS PCI optimization criteria) result, the target vessel failure rate was only 2.5% during the one-year follow-up. This finding is similar to other trials, which used IVUS to guide PCI. The ULTIMATE trial demonstrated a TVF rate of 2.9% in the IVUS group (vs. 5.4% in the angiography group); the IVUS-XPL trial showed 2.9% in the IVUS group (vs. 5.8% in the angiography group); and Kim et al.’s study showed 4.0% in the IVUS group (vs. 8.1% in the angiography group) during a 12-month follow-up. In all these trials, the stented segment was significantly shorter compared to our study; thus, our findings provide reassurance that even very long coronary artery lesions can be treated with a satisfactory short-term TVF rate.

We believe that our data could be beneficial in filling the gaps in the knowledge regarding very long coronary artery lesion treatment. Our study’s results could supplement the existing evidence and encourage operators that with intravascular imaging guidance, even very long coronary artery lesions can be treated percutaneously without leaving ischemia behind and with an acceptable negative events rate. 

## 5. Limitations

The results of our study, however, should be interpreted in a view of certain limitations. First, this is a single-arm prospective, non-blinded trial, thus we cannot compare our results to angiography-guided PCI; however, data from previous studies have already demonstrated the benefit of IVUS and FFR when compared to angiography. The sample size is relatively small, therefore, due to low event rates, we could not ascertain the prognostic factors of TVF. The follow-up duration is one year, however; these are initial results and we will continue to observe our sample, and the results of a longer follow-up will be published in the future. We believe that the results of our trial should encourage the development of a randomized controlled trial where both FFR and IVUS are used in the PCI of complex coronary artery lesions and compared to CABG.

## 6. Conclusions

After the percutaneous treatment of very long coronary artery lesions, both optimal functional and anatomical results were achieved only in a minority of patients, which underlines the challenges related to these complex lesions. However, the strategy combining FFR (to assess baseline ischemia and evaluate functional PCI result) and essentially IVUS (to optimize the procedure) was associated with a low one-year TVF rate and no residual myocardial ischemia after PCI in very long coronary artery lesions.

## Figures and Tables

**Table 1:** Comparison of clinical characteristics between post-PCI FFR ≥ 0.9 and < 0.9 patient groups.

Characteristic	All (*n* = 80)	FFR ≥ 0.9 (*n* = 30)	FFR < 0.9 (*n* = 50)	*p*
Age, years	66.2 ± 8.9	65.9 ± 9.2	66.4 ± 9.0	0.83
Male sex	57 (71.3)	22 (73.3)	35 (70.0)	0.75
Diabetes	15 (18.8)	6 (20.0)	9 (18.0)	0.82
Hypertension	74 (92.5)	27 (90.0)	47 (94.0)	0.51
Dyslipidemia	72 (91.1)	26 (89.7)	46 (92.0)	0.72
Chronic kidney disease	16 (20.0)	6 (20.0)	10 (20.0)	1
Active smoking	20 (25.0)	9 (30.0)	11 (22.0)	0.42
History of non-index vessel PCI	47 (58.8)	18 (60.0)	29 (58.0)	0.86
History of CABG	1 (1.3)	1 (3.3)	0 (0.0)	0.38
Previous MI	46 (57.5)	18 (60.0)	28 (56.0)	0.73
Indications for PCI				
Stable angina	30 (37.5)	10 (33.3)	20 (40.0)	0.58
Unstable angina	7 (8.8)	2 (6.7)	5 (10.0)	
NSTEMI	13 (16.3)	5 (16.7)	8 (16.0)	
Silent ischemia	4 (5.0)	3 (10.0)	1 (2.0)	
Staged PCI after STEMI	26 (32.5)	10 (33.3)	16 (32.0)	
LV ejection fraction, %	52.0 ± 5.2	51.5 ± 5.0	52.3 ± 5.3	0.27
Hemoglobin, g/dl	139.7 ± 15.6	141.0 ± 14.9	138.8 ± 16.2	0.55
Creatinine, µmol/L	85.5 ± 22.0	81.5 ± 21.4	87.9 ± 22.3	0.21
Total cholesterol, mmol/l	5.2 ± 1.6	5.0 ± 1.3	5.2 ± 1.8	0.66
LDL, mmol/l	3.3 ± 1.3	3.3 ± 1.1	3.4 ± 1.4	0.74

PCI—percutaneous coronary intervention; CABG—coronary artery bypass graft surgery; MI—myocardial infarction; NSTEMI—myocardial infarction without ST-segment elevation; STEMI—myocardial infarction with ST-segment elevation; LV—left ventricle; LDL—low-density lipoprotein.

**Table 2:** Comparison of procedural characteristics between post PCI FFR ≥ 0.9 and < 0.9 patient groups.

Characteristic	All (*n* = 80)	FFR ≥ 0.9 (*n* = 30)	FFR < 0.9 (*n* = 50)	*p*
No of diseased vessels				
1	12 (15.0)	4 (13.3)	8 (16.0)	0.91
2	35 (43.8)	14 (46.7)	21 (42.0)	
3	33 (41.3)	12 (40.0)	21 (42.0)	
Target vessel				
LAD	66 (82.5)	16 (53.3)	50 (100.0)	0.0001
LCx	7 (8.8)	7 (23.3)	0 (0.0)	
RCA	7 (8.8)	7 (23.3)	0 (0.0)	
Successful PCI	80 (100.0)	30 (100.0)	50 (100.0)	
Predilatation	80 (100.0)	30 (100.0)	50 (100.0)	
Largest predilation balloon diameter, mm	2.7 ± 0.3	2.7 ± 0.3	2.7 ± 0.3	0.91
Maximal predilation pressure, atm	15.4 ± 2.2	15.3 ± 1.9	15.4 ± 2.4	0.99
Number of stents implanted	1.85 ± 0.6	1.87 ± 0.6	1.84 ± 0.6	0.85
Average stent implantation pressure, atm	12.2 ± 1.6	12.7 ± 1.7	11.9 ± 1.4	0.03
Average stent diameter, mm	3.3 ± 0.4	3.4 ± 0.4	3.2 ± 0.3	0.14
Total stent length, mm	62.3 ± 18.0	61.8 ± 19.9	62.5 ± 17.0	0.85
Stent length ≥ 50 mm	53 (63.3)	17 (56.7)	36 (72.0)	0.16
Postdilatation	80 (100.0)	30 (100.0)	50 (100.0)	
Largest postdilation balloon, mm	4.2 ± 0.5	4.0 ± 0.5	4.2 ± 0.5	0.07
Maximal balloon pressure, atm	17.8 ± 2.9	18.3 ± 3.2	17.4 ± 2.7	0.09
Bifurcation two-stent technique	7 (8.8)	1 (3.3)	6 (12.0)	0.25
Procedure time, min	77.4 ± 27.7	78.2 ± 39.7	76.9 ± 17.1	0.32
Contrast volume, ml	157.7 ± 41.4	150.7 ± 45.6	162.0 ± 38.4	0.18

LAD—left anterior descending artery; LCx—left circumflex artery; RCA—right coronary artery; PCI—percutaneous coronary intervention.

**Table 3:** Comparison of fractional flow reserve measurement characteristics between post-PCI FFR ≥ 0.9 and < 0.9 patient groups.

Characteristic	All (*n* = 80)	FFR ≥ 0.9 (*n* = 30)	FFR < 0.9 (*n* = 50)	*p*
FFR pre-PCI	0.64 ± 0.1	0.64 ± 0.12	0.64 ± 0.09	0.75
FFR post-PCI	0.89 ± 0.05	0.94 ± 0.04	0.86 ± 0.02	0.0001
Distal gradient	0.04 ± 0.03	0.02 ± 0.02	0.05 ± 0.03	0.0001
Trans-stent gradient	0.07 ± 0.03	0.04 ± 0.02	0.08 ± 0.02	0.0001

FFR—fractional flow reserve; PCI—percutaneous coronary intervention.

**Table 4:** Comparison of intravascular ultrasound characteristics between post-PCI FFR ≥ 0.9 and < 0.9 patient groups.

Characteristic	All (*n* = 80)	FFR ≥ 0.9 (*n* = 30)	FFR < 0.9 (*n* = 50)	*p*
Number of IVUS runs				
2	50 (62.5)	19 (63.3)	31 (62.0)	0.98
3	27 (33.8)	10 (33.3)	17 (34.0)	
4	3 (3.8)	1 (3.3)	2 (4.0)	
Distal reference EEM diameter, mm	3.3 ± 0.5	3.4 ± 0.5	3.2 ± 0.5	0.12
Proximal reference EEM diameter, mm	4.6 ± 0.5	4.6 ± 0.5	4.7 ± 0.5	0.28
Minimal lumen diameter, mm	1.8 ± 0.2	1.8 ± 0.2	1.8 ± 0.2	0.93
Minimal lumen area, mm^2^	2.5 ± 0.6	2.6 ± 0.7	2.5 ± 0.6	1
Calcium arc ≥ 180°	39 (48.8)	13 (43.3)	26 (52.0)	0.45
Distal reference lumen area, mm^2^	5.9 ± 1.9	6.5 ± 2.1	5.5 ± 1.7	0.03
Distal reference EEM area, mm^2^	8.9 ± 3.3	9.9 ± 3.6	8.3 ± 3.1	0.06
Distal reference plaque burden, %	32.6 ± 9.2	32.1 ± 9.6	32.9 ± 9.1	0.72
Proximal reference lumen area, mm^2^	10.5 ± 2.8	10.6 ± 2.6	10.5 ± 3.0	0.7
Proximal reference EEM area, mm^2^	18.2 ± 4.1	18.0 ± 4.6	18.4 ± 3.9	0.76
Proximal reference plaque burden, %	42.0 ± 8.4	40.4 ± 6.5	42.9 ± 9.2	0.28
Minimal stent diameter, mm	2.5 ± 0.4	2.6 ± 0.4	2.5 ± 0.4	0.25
Minimal stent area, mm^2^	5.9 ± 1.9	6.3 ± 1.8	5.6 ± 1.8	0.12
Good stent expansion	73 (92.4)	26 (86.7)	47 (95.9)	0.13
Good stent apposition	79 (100.0)	30 (100.0)	49 (100.0)	
No stent edge dissection	79 (100.0)	30 (100.0)	49 (100.0)	
Plaque ≤ 50% near stent edges	56 (70.9)	21 (70.0)	35 (71.4)	0.89
Optimal IVUS result	54 (68.4)	20 (66.7)	34 (69.4)	0.8

IVUS—intravascular ultrasound; EEM—external elastic membrane.

**Table 5:** Medications at discharge comparison between post-PCI FFR ≥ 0.9 and < 0.9 patient groups.

Medication	All (*n* = 80)	FFR ≥ 0.9 (n = 30)	FFR < 0.9 (*n* = 50)	*p*
DAPT	71 (88.8)	26 (86.7)	46 (90.0)	0.65
OAC and antiplatelet	9 (11.2)	3 (10.0)	6 (12.0)	0.84
Statin	74 (92.5)	28 (93.3)	46 (92.0)	1
Beta-blocker	67 (83.8)	24 (80.0)	43 (86.0)	0.48
ACE-i/ARB	68 (85.0)	26 (86.7)	42 (84.0)	0.75

DAPT—double antiplatelet therapy; OAC—oral anticoagulant; ACE-I—angiotensin-converting enzyme inhibitor; ARB—angiotensin receptor blocker.

**Table 6:** Adverse events during the 12-month follow-up.

Adverse Event	All Patients
Target-vessel-related death	0 (0)
Target-vessel-related myocardial infarction	0 (0)
Target-vessel ischemia-driven revascularization	2 (2.5)
Target vessel failure	2 (2.5)
Cardiac death	1 (1.25)
All-cause death	1 (1.25)

Adverse Event

All Patients

## Data Availability

All data can be shared by the authors upon reasonable request and in accordance with Lithuanian privacy regulations.
